# An Improved Air Health Index Based on Short-Term Cardiovascular Effects in Tianjin, China

**DOI:** 10.3389/ijph.2024.1607214

**Published:** 2024-09-16

**Authors:** Mengnan Zhang, Yu Bai, Junyi Hu, Yang Ni, Qiang Zeng

**Affiliations:** ^1^ Tianjin Centers for Disease Control and Prevention, Tianjin, China; ^2^ Huaian Center for Disease Control and Prevention, Huaian, China

**Keywords:** air pollution, ambient temperature, air health index, years of life lost, cardiovascular

## Abstract

**Objectives:**

To construct an improved air health index (AHI) based on cardiovascular years of life lost (YLL) in Tianjin and assess its utility.

**Methods:**

We derived the exposure-response coefficients from time-series models and calculated the excess YLL (EYLL) for simultaneous exposure to air pollution and non-optimum temperature. The AHI was developed using the EYLL at the WHO 2021 Air Quality Guideline annual mean values and optimum temperature as a reference. We assessed the validity of AHI by comparing the correlations and model fit between the AHI, air quality health index (AQHI), and air quality index (AQI) with cause-specific YLLs.

**Results:**

Each inter quartile range (*IQR*) increase in AHI was associated with 256.31 (95%*CI*: 183.05, 329.57), 150.34 (95%*CI*: 108.23, 192.46), 90.41 (95%*CI*: 64.80, 116.02) and 60.80 (95%*CI*:33.41, 88.18) person-year increments for non-accidental, cardiovascular, ischaemic, and cerebrovascular YLL, respectively. The AHI, in contrast to the AQHI and AQI, showed the strongest correlations with the risks of cause-specific YLLs, both in the total population and subpopulations.

**Conclusion:**

The AHI based on cardiovascular YLL has a greater predictive ability for health risks.

## Introduction

In the context of global climate change, both air pollution and non-optimum ambient temperature have been recognized as serious environmental risk factors for human health, such as cardiovascular diseases and respiratory diseases [1–4]. The Global Burden of Disease Study in 2019 reported that air pollution was accountable for over 7 million deaths and tens of millions of disability-adjusted life years worldwide, with roughly 1.85 million untimely deaths and 42.51 million disability-adjusted life years resulting from air pollution exposure in China [5]. It has been estimated that non-optimum ambient temperature was responsible for 1.01 million deaths among men and 946 thousand deaths among women globally [5]. In addition, several studies have shown that air pollution and non-optimum ambient temperature could trigger comparable biological mechanisms in the induction of adverse health impacts, such as systemic inflammatory pathways, oxidative stress, and immune function [6, 7]. Therefore, simultaneously exposure to air pollution and non-optimum temperature may lead to greater health risks and burden of diseases, so that it is necessary to construct an indicator that could comprehensively assess the health risks of exposure to air pollution and non-optimum ambient temperature simultaneously.

At present, the air quality index (AQI) and air quality health index (AQHI) have been used in many countries and regions around the world to inform the public about the health risks associated with air pollution [8–10]. However, there are several limitations of these indicators. Firstly, as the AQI is a simple and easy-to-understand numeric scale ranging from 0 to 500 determined by the pollutant with the highest subindex, AQI may ignore other pollutants except the main pollutant, which means that it is unable to capture the cumulative influence of multiple air pollutants. Otherwise, the AQI fails to reflect the no-threshold exposure-response relationships between pollution and health outcomes. AQHI is an improved indicator which is constructed as the sum of the excess health risks associated with individual pollutants or mixtures of pollutants [11]. Several previous studies have constructed the AQHI and assessed its validity with the result that the AQHI is a better predictive indicator of health risks than the AQI [10, 12–15]. However, the current AQHI was established without taking the health effects of ambient non-optimum temperature into account, so it may lead to the underestimation while predicting the public health risks.

To address the limitation, the air health index (AHI) was first proposed in China by Professor Kan Haidong’s team [16]. This team, using a nationwide database, established the exposure-response relationships of major air pollutants and non-optimum ambient temperature with mortality, and constructed corresponding AHI by combining the excess health risks associated with air pollutants and non-optimum temperature on mortality. This study showed that AHI exhibited a good performance in predicting the daily mortality risks [16]. However, this study also pointed out two limitations of the AHI. Firstly, the predictability of the AHI may be less stable over a wide area, such as a country, than over a small region with homogenous atmospheric and demographic characteristics. In addition, it remains unclear whether AHI is a better indicator in predicting other health risks like cardiovascular diseases. The short-term effects of air pollution and ambient temperature on cardiovascular diseases have been studied in many regions [4, 17–19]. The statistically significant associations of air pollution and non-optimum temperature with cardiovascular diseases also were found in Tianjin [20, 21].

In addition, current AHI was constructed using mortality as a substitute for human health which giving equal weight to each death without taking into account the effects of age at death may result in a bias in forecasting air quality and health risks highlighted by the AHI. In contrast, years of life lost (YLL), which can take premature death and life expectancy into consideration and has been widely used as an indicator of health outcomes in the assessment of the effects of air pollution and ambient temperature [22, 23]. Therefore, in this study we chose cardiovascular YLL as an indicator of outcomes to establish an urban AHI in Tianjin by taking consideration of the effects of exposure to air pollution and non-optimum ambient temperature simultaneously.

## Methods

### Data Sources

The Tianjin Environmental Monitoring Center provided the daily average concentrations of PM_2.5_, PM_10_, SO_2_, NO_2_, CO, and O_3_, as well as daily AQI monitoring data, from 1st January 2014 to 31st December 2017 for six urban districts of Tianjin.

The daily average ambient temperature and relative humidity for the same period in the six urban districts of Tianjin were collected from the Tianjin Meteorological Bureau.

Data on mortality were collected from the Death Register and Report Information System of the Tianjin Centers for Disease Control and Prevention. We assessed a range of causes of mortality based on the sole primary diagnosis coded by the International Statistical Classification of Disease and Related Health Problems, 10th Revision (ICD-10), including non-accidental causes (A00-R99), cardiovascular diseases (I00-I99), ischaemic heart diseases (I20-I25), and cerebrovascular diseases (I60-I69). The cause-specific YLLs were calculated using the formula according to the WHO’s standard life table formula for YLLs. The formula has been described in detail previously [24]. The cardiovascular YLL was used as the health outcome to evaluate the exposure-response relationships of air pollution and non-optimum ambient temperature and to construct the AHI. The other cause-specific YLLs were used for the assessment of the validity of the AHI.

### Estimating the Associations of Air Pollution With Cardiovascular YLL

The generalized additive models (GAM) and weighted quantile sum models (WQS) were fitted to establish the exposure-response relationship between air pollution and cardiovascular YLL.

First, single-pollutant GAMs were constructed to assess the exposure-response relationships between the six major air pollutants and cardiovascular YLL at different lag days. This aimed to identify the air pollutants that caused adverse effects and the lag days with the greatest health effects. The model is shown in Equation 1.

$$E\left({YLL}_{t}\right)=\alpha +\beta X_{i,t-l}+ns\left(trend,7*4\right)+s\left({temp}_{lag03}\right)+s\left({rh}_{lag03}\right)+Dow+Holiday$$ (1)

Where *E(YLL*_*t*_ *)* represents the anticipated quantity of cardiovascular YLL on day *t*; *α* denotes the intercept; *X*_*i,t-l*_ is the concentration of air pollutant *i* on lag day *l*, taking into account single lags from the current day to the previous 3 days (lag0 to lag3), as well as cumulative lags from the current day to the previous 3 days (lag01 to lag03); *β* represents the exposure-response coefficients; *ns(trend*, 7*4) denotes a natural spline function with degree *7/year* to control for the non-linear confounding effects of long-term and seasonal trends; *temp*_*lag03*_ and *rh*_*lag03*_ are the average values for average ambient temperature and average relative humidity from lags 0 to 3, *s(temp*_*lag03*_ *)* and *s(rh*_*lag03*_ *)* are penalized spline functions to control potential confounding effect of average ambient temperature and relative humidity, respectively [25]; *DOW* and *Holiday* are the dummy variables to account for the short-term cyclic fluctuations in the data.

Secondly, we determined air pollutants mixture according to the outcomes of the single-pollutant GAMs mentioned above and employed the WQS to examine the exposure-response relationship between air pollutants mixture and cardiovascular YLL. The model is shown in Equation 2.

$$E\left({YLL}_{t}\right)=\alpha +\beta \sum_{i=1}^{p}w_{i}X_{i}+ns\left(trend,7*4\right)+s\left({temp}_{lag03}\right)+s\left({rh}_{lag03}\right)+Dow+Holiday$$ (2)

Where *β* indicates the type number of air pollutants, with a maximum value of six; The $\sum_{i=1}^{p}w_{i}X_{i}$ term represents the air pollutants mixture, *W*_*i*_ is the unknown weight of air pollutant *i* in the air pollutants mixture, *X*_*i*_ indicates the concentration of pollutant *i* on the lag day of the greatest health effect; Other variables are consistent with the above formula.

To estimate the model, the data were split into a training and a validation dataset. The former was utilized for weight estimation whilst the latter was used to test the significance of the final WQS index. The weights were assessed using a bootstrap approach and were restricted to sum to one and to be bounded between zero and one ( $\sum_{i=1}^{p}w_{i}=1$ , 0≤*w*_*i*_ ≤ 1) [26, 27].

### Estimating the Associations of Non-Optimum Ambient Temperature With Cardiovascular YLL

Our initial analysis, as presented in the Supplementary Material, using the distributed lag nonlinear model (DLNM) indicated a U-shaped relationship between ambient temperature and cardiovascular YLL in Tianjin, with possible cold and hot thresholds. Therefore, we constructed a double-threshold distributed lag linear model (DLM), which assumes that the impact of low temperature follows a linear pattern below the cold threshold, and the impact of high temperature is linear above the hot threshold. The model is shown in Equation 3.

$$E\left({YLL}_{t}\right)=\alpha +\beta_{c}TC+\beta_{h}TH+\sum ns\left(X,3\right)+ns\left(trend,7*4\right)+s\left({rh}_{lag03}\right)+Dow+Holiday$$ (3)

Where *TC* and *TH* are matrices obtained by applying the double-threshold DLM to ambient temperature below the cold threshold and above the hot threshold respectively, the method for determining *TC* and *TH* has been described in detail in a previous study [28]; *X* are the air pollutants (the two air pollutants with the highest weight in the WQS), *ns(X,3)* is a non-parametric smoothing function of degree *3* to control for the confounding effect of air pollutants.

### Constructing of AHI

Based on these exposure-response relationships obtained above, we calculated the daily excess YLL (EYLL) due to exposure to air pollution and non-optimum ambient temperature. The reference value of zero was utilized to calculate the excess risk associated with air pollutants. As air pollutants concentrations are standardized in the evaluation process of the WQS, it is essential that the reference value of zero is also standardized to prevent the occurrence of negative values in the calculation of the excess health risk. To calculate the excess health risk of non-optimum ambient temperature, cold or hot thresholds were used as a reference.

Then, using the annual standard values of each pollutant specified in the WHO 2021 Air Quality Guideline and optimum ambient temperature range as reference, the daily EYLL was indexed to a scale of 0–10+ to construct the AHI. Specifically, index value 3 corresponds to the EYLL derived from air pollution and ambient temperature that meet the annual standard values of the WHO 2021 Air Quality Guideline and the optimal ambient temperature range respectively. To evaluate the reliability of the AHI effectively, we also developed the AQHI, which takes into account only health risks caused by air pollution. And we categorized the AHI as low (0–3), moderate (4–6), and high (7–10+), based on prior research on air quality index grading [21].

### Validity Evaluation of AHI

We assessed the validity of the AHI in predicting health risks by comparing the associations of the AHI, AQHI, and AQI with various health outcomes, including non-accidental, cardiovascular, cerebrovascular, and ischaemic YLL. The relationships between these three indexes and health outcomes in different subgroups were also compared. Because of the different numerical scales of these three indexes, AHI (0–10+), AQHI (0–10+), and AQI (0–500+), we used the inter quartile range (*IQR*) with increasing AHI, AQHI and AQI to assess the ability of the indices to predict health risks.

## Results

### Descriptive Analysis

(Supplementary Table S1) summarized the air pollution, meteorological factors, and health outcomes in Tianjin during the study period. The daily average concentrations of PM_2.5_, PM_10_, SO_2_, NO_2_, CO, and O_3_ were 71.97 μg/m^3^, 116.19 μg/m^3^, 28.02 μg/m^3^, 47.88 μg/m^3^, 1,606.44 μg/m^3^, and 58.04 μg/m^3^, respectively. The mean AQI was 105.45. The daily average temperature and average relative humidity were 13.94°C and 55.37%, respectively. The daily average non-accidental, cardiovascular, cerebrovascular, and ischaemic YLL were 1790.19, 1,102.27, 454.44, and 532.51 person-year, respectively.

### The Associations of Air Pollution With Cardiovascular YLL

The results of the single-pollutant GAMs showed that PM_10_, SO_2_, NO_2_, and O_3_ had statistically significant effects on cardiovascular YLL in Tianjin (*P* < 0.05), and the maximum effect of each pollutant all occurred at the cumulative lag day (Supplementary Table S2). Specifically, the maximum health effects of PM_10_, NO_2_, and O_3_ occurred on lag01 with the effect values of 1.82 (95%*CI*: 0.01, 3.63), 6.99 (95%*CI*: 0.15, 13.82), and 4.34 (95%*CI*: 0.89, 7.81) respectively. The maximum effect of SO_2_ occurred on lag02 with the effect value of 12.91 (95%*CI*: 4.50, 21.31). Therefore, PM_10_, NO_2_, and O_3_ at lag01, and SO_2_ at lag02 were selected as the air pollutants mixtures for the subsequent analysis.

The result of the WQS was presented in Table 1, which showed the relative weights of each air pollutant in the air pollutants mixture, and the exposure-response relationship between the air pollutants mixture and cardiovascular YLL. SO_2_ had the largest weight of 0.447, followed by O_3_ and PM_10_ with relative weights of 0.327 and 0.179 respectively, and NO_2_ had the smallest of 0.046. It suggested that per unit increase in the concentrations of SO_2_, O_3_, and PM_10_ would result in greater health risks than NO_2_ in the air pollutants mixture. And the result revealed that the air pollutants mixture was also associated with an increased health risk of cardiovascular YLL. Each unit increase in the air pollutants mixture was associated with an increase of 30.41 person-year of cardiovascular YLL.

**TABLE 1:** The exposure-response relationship between air pollutants mixture and cardiovascular years of life lost in Tianjin, 2014–2017.

Air pollution	Weight	*β*	*SE*	*P*
PM_10_	0.179	30.408	15.365	0.048
SO_2_	0.447			
NO_2_	0.046			
O_3_	0.327			

PM_10_, NO_2_, and O_3_ were the concentrations at lag01, SO_2_ was the concentration at lag02; Weight: the relative weights of each air pollutant in the air pollutants mixture; *β*: the exposure-response of the air pollutants mixture and cardiovascular years of life lost; *SE*: standard error.

### The Associations of Non-Optimum Ambient Temperature With Cardiovascular YLL

The DLNM showed a U-shaped relationship between average ambient temperature and cardiovascular YLL, with a large comfortable temperature range where the average ambient temperature has no statistically adverse effect on cardiovascular YLL. And the result indicated that the possible cold threshold was between −3.0°C and 3.0°C and the possible hot threshold was between 18°C and 23°C (Supplementary Figure S1). Therefore, multiple double-threshold DLMs were fitted to determine the combination of cold threshold (from −3.0°C to 3.0°C) and hot threshold (from 18.0°C to 23.0°C) with minimum residual deviation. We found that the cold and hot thresholds for cardiovascular YLL were 0°C and 19.0°C respectively (Supplementary Table S3).

**FIGURE 1:**
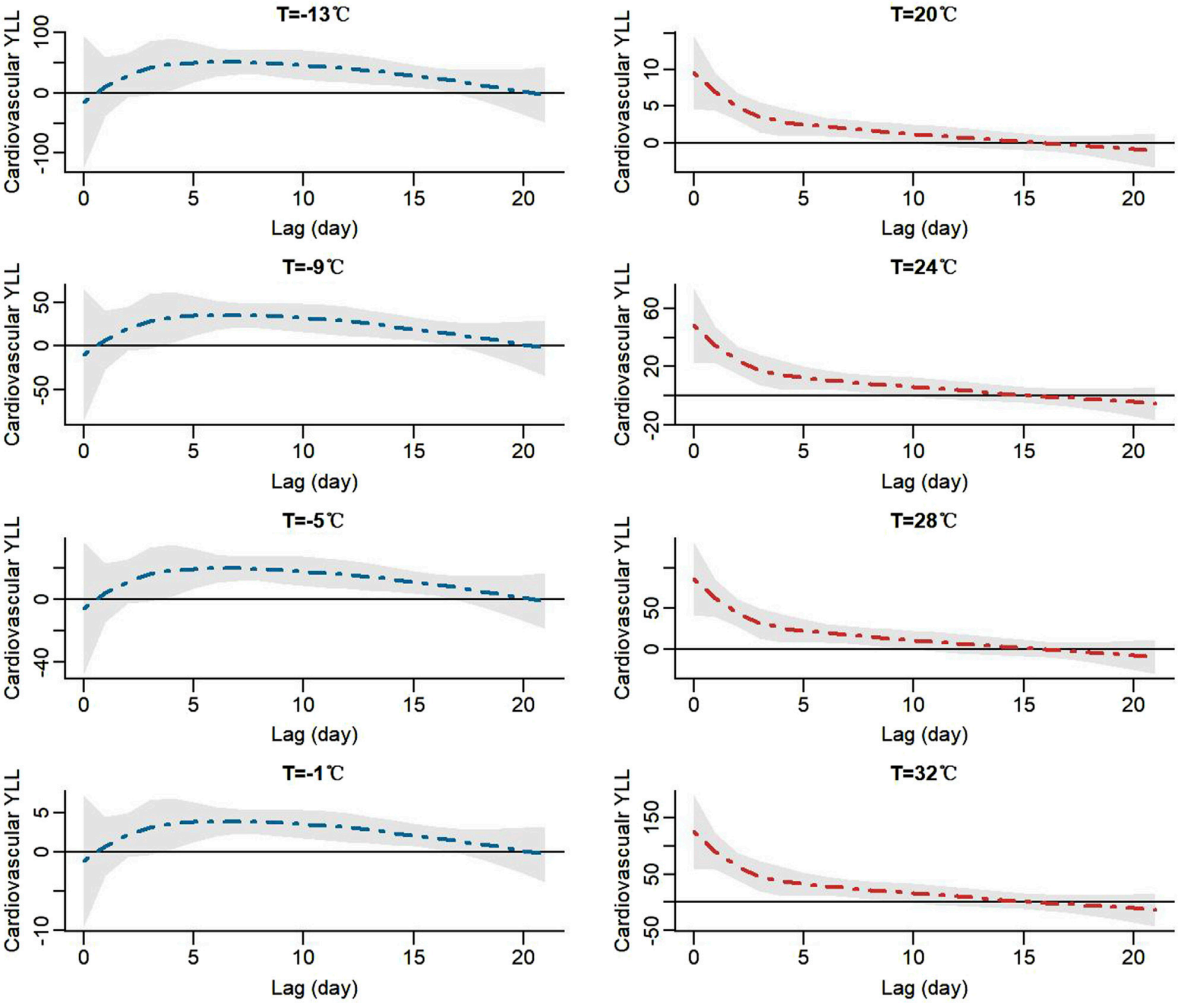
The lag exposure-response relationship of low and high ambient temperature with cardiovascular years of life lost in Tianjin, 2014–2017, using a double-threshold distributed lag linear model.

Figure 1, basing a double-threshold DLM with 0°C as the cold threshold and 19°C as the hot threshold, showed the lag exposure-response relationships of low and high ambient temperature on cardiovascular YLL. Results showed that a significant cold effect on cardiovascular YLL appeared after a delay of 3 days and lasted for 18 days, whereas a significant hot effect of cardiovascular YLL occurred within 0–10 days. We calculated the overall effects of cold and hot on cardiovascular YLL along the lags (Table 2). For cold effect over lag 0–18 days, a 1°C decrease in ambient average temperature below the cold threshold was associated with a 44.68 (95%*CI*: 24.25, 65.11) person-year increase in cardiovascular YLL. For hot effect over lag 0–10 days, a 1°C increase in ambient average temperature above the hot threshold was associated with a 36.27 (95%*CI*: 22.83, 49.71) person-year increase in cardiovascular YLL.

**TABLE 2:** The cumulative cold and hot effects of ambient temperature on cardiovascular years of life lost in Tianjin, 2014–2017.

Effect	Lag (days)	Estimates (95%CI)
Cold effect	0–18	44.68 (24.25–65.11)*
Hot effect	0–10	36.27 (22.83–49.71)*

The cold effect is the increase in cardiovascular years of life lost for a 1°C ambient temperature decreases below the cold threshold (0°C); The hot effect is the increase in cardiovascular years of life lost for a 1°C ambient temperature increases beyond the hot threshold (19°C); *P < 0.05.

### The Calculation and Validation of AHI

The mean AHI was 15.60, the minimum and maximum AHIs were 2.47 and 44.61, respectively, and the *IQR* of AHI was 18.15. The mean AQHI was 6.12, the minimum and maximum AQHIs were 1.72 and 17.03, respectively, and the *IQR* of AQHI was 2.23. The mean AQI was 105.45, the minimum and maximum AQIs were 19.36 and 436.96, respectively, and the *IQR* of AQI was 63.09 (Supplementary Table S3).

**FIGURE 2:**
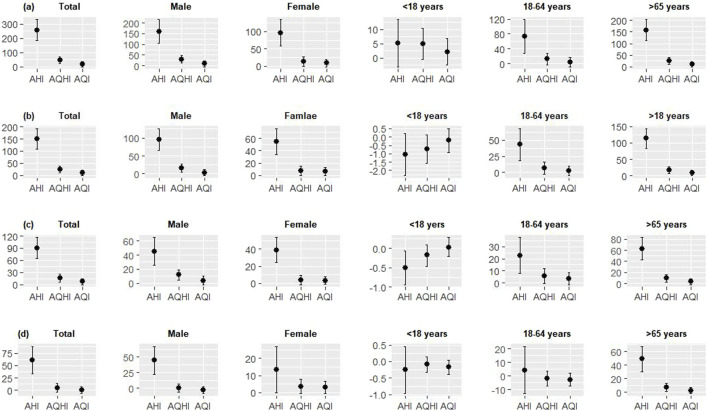
The relationships of per inter quartile range increase in air health index, air quality health index, and air quality index with daily non-accidental **(A)**, cardiovascular **(B)**, ischaemic **(C)**, and cerebrovascular **(D)** years of life lost in the total population and subpopulations in Tianjin, 2014–2017.

Table 3 displayed the health risks of AHI, AQHI, and AQI associated with different health outcomes. AHI and AQHI were associated with increased risks of cause-specific YLLs, while no significant associations were observed for AQI. Each *IQR* increase in AHI was associated with 256.31 (95%*CI*: 183.05, 329.57), 150.34 (95%*CI*: 108.23, 192.46), 90.41 (95%*CI*: 64.80, 116.02), and 60.80 (95%*CI*: 33.41, 88.18) person-year increments for non-accidental, cardiovascular, ischaemic, and cerebrovascular YLL, respectively, whereas the increments were 45.42 (95%*CI*: 21.43, 69.41), 23.78 (95%*CI*: 9.13, 38.43), 15.93 (95%*CI*: 6.33, 25.52), and 4.88 (95%*CI*: 3.82, 13.59) for each unit increase in AQHI, respectively. And the AHI generally showed the strongest associations with the risk of cause-specific YLL, either in the total population or in subpopulations, compared with the AQHI and AQI (Figure 2).

**TABLE 3:** The relationships of air health index, air quality health index, and air quality index with daily cause-specific years of life lost in Tianjin, 2014–2017.

YLLs	AHI			AQHI			AQI		
	Estimates	R^2^	GCV	Estimates	R^2^	GCV	Estimates	R^2^	GCV
Non-accidental	256.31* (183.05, 329.57)	0.262	90,740	45.42* (21.43, 69.41)	0.246	92,922	19.09 (−0.47, 38.64)	0.240	93,606
Cardiovascular	150.34* (108.23, 192.46)	0.294	33,795	23.78* (9.13, 38.43)	0.263	34,478	10.09 (−1.83, 22.01)	0.260	34,655
Ischaemic	90.41* (64.80, 116.02)	0.249	14,407	15.93* (6.33, 25.52)	0.236	14,692	7.30 (−0.48, 15.07)	0.232	14,767
Cerebrovascular	60.80* (33.41, 88.18)	0.132	12,447	4.88 (−3.82, 13.59)	0.124	12,616	0.19 (−6.91, 7.28)	0.123	12,626

Estimates are presented as percentage changes and 95%CI, associated per IQR, increase in indices; R2 is the coefficient of determination and GCV, is the generalized cross-validation value; *P < 0.05.

The classification of AHI and health messages associated with each category of the index are shown in Table 4. 0–3 was classified as “low,” 4–6 as “moderate,” and 7–10+ as “high” marked with yellow, orange, and red, respectively. The primary health messages are to reduce the intensity and duration of outdoor activities, and to safeguard proper temperature conditions, for instance, by using an air conditioning system and dressing appropriately for the prevailing weather conditions.

**TABLE 4:** Classification and health messages of air health index in Tianjin, 2014–2017.

Risk levels	EYLL	AHI	Health messages	
			Health implications	Cautionary statements
low	<32.186	0–3	Air quality and temperature are satisfactory, and air pollution and temperature present minimal to no risk.	minimal to no risk
moderate	32.186∼64.372	4–6	Air quality and temperature are within acceptable levels. However, there might be a moderate health concern for a small number of individuals who are extremely sensitive to air pollution or temperature.	If you have heart or breathing problems and exhibit symptoms, you might contemplate reducing physical activity outside or/and safeguarding proper temperature conditions.
high	>64.372	7–10+	Some members of the general public may experience health effects; members of sensitive groups may experience more severe health effects.	Anyone who experiences discomfort, such as a cough or sore throat, should consider reducing strenuous outdoor activities or/and safeguarding proper temperature conditions.

## Discussion

The AQI is a widely used index to characterize air quality and its health risks worldwide. It classifies air quality into classes based on the pollutant with the highest subindex (the top pollutant) with the disadvantage of not reflecting the additive health effects of multiple air pollutants. To address the shortcomings of the AQI, Environment Canada and Health Canada in 2008 proposed the AQHI, an index constructed based on the concentrations of multiple air pollutants and the exposure-response relationships between these air pollutants and health outcomes [11]. This provides the AQHI with a good performance in predicting the health risk of ambient air pollution.

However, within the context of global climate change, non-optimum ambient temperature is also a substantial environmental risk factor for human health. As a result, it is crucial to create an index that precisely reflects the health risks associated with non-optimum ambient temperature. Current indices measuring the health risks related to ambient temperature, such as the heat wave index and cold wave index, only account for extreme ambient temperature, neglecting the considerable health risks associated with moderate non-optimum temperature. Thus, the heat wave and cold wave indexes could significantly underestimate the health risks associated with non-optimum ambient temperature. The AHI incorporates the assessment of the exposure-response relationship between non-optimum temperature and health, as per the AQHI construction strategy [16]. This approach comprehensively reflects the health hazards linked to exposure to multiple ambient pollutants, along with effectively communicating the health risks of exposure to non-optimal temperature, which is highly significant for protecting public health.

In this study, we established the Tianjin AHI by integrating the health risks of ambient air pollution and non-optimum temperature with cardiovascular YLL and evaluated its validity. To the best of our knowledge, this study is the inaugural attempt at formulating a regional AHI whilst simultaneously utilizing cardiovascular YLL as a health outcome indicator. China is a large country with significant variations in atmospheric pollution, meteorological conditions, and population susceptibility across different regions. As a result, the predictability of AHI may be less stable in larger regions, such as the entire country, where atmospheric and population characteristics are less homogeneous. Furthermore, current health risk assessments require further differentiation of the disease based on health-risk groups [29, 30]. Short-term exposure to air pollution and non-optimum temperature could usually cause various diseases, mainly cardiovascular diseases and respiratory diseases [31–34]. Moreover, certain studies have demonstrated variations in the accuracy of air quality indices when evaluating diverse health risk groups [35]. It is therefore necessary to explore new indices that reflect the combined health risks of air pollution and non-optimal temperatures for different health risk groups, such as those with cardiovascular disease.

In addition, WQS was used to assess the exposure-response relationship between simultaneous exposure to major air pollutants and cardiovascular YLL in this study. WQS, developed explicitly for the context of environmental mixture analysis, is an increasingly widespread approach that enables the assessment of the association between a mixture and an outcome by generating a summary score of the mixture in a supervised manner [25, 26]. Because individuals are typically exposed to multiple simultaneously, and multiple air pollutants are often highly correlated with one another, WQS can more precisely evaluate the health risks of combined exposures to multiple air pollutants, compared to the traditional use of single-pollutant and two-pollutant models.

Furthermore, if Tianjin AHI is constructed according to Team Kan’s strategy, the AHI on any given day will be related to the day with the maximum pollution and non-optimal temperature observed during the 4 years in Tianjin. That arbitrary point of reference is not only irrelevant for the rest of the world but sooner or later also for the city of Tianjin. An index should have some generalizable dimension. Adebajo et al recently provided an intriguing point of reference to build a globally generalizable framework of air quality index, namely the WHO 2021 Air Quality Guideline annual mean values [21]. Therefore, we attempted to reconstruct the AHI using the WHO 2021 Air Quality Guideline annual mean values as a reference. The results showed that the majority of AHI is classified as “high risk” when using the WHO 2021 Air Quality Guideline annual mean values as a reference to construct the Tianjin AHI, with almost none falling under the “low risk” category. This indicates the necessity for further intensification of efforts to address air pollution and climate change in Tianjin, with the objective of protecting public health and reducing the associated disease burden.

Results demonstrated notable correlations between the AHI and non-accidental, cardiovascular, ischaemic, and cerebrovascular YLL, all of which surpassed those of the AQHI. No statistically significant correlations between AQI and these health outcomes were found. The GCV values for the AHI model were consistently lower than those for the AQHI and AQI models. Furthermore, the R^2^ values of the AHI model were uniformly higher when compared to the R^2^ values of the AQHI and AQI models. These findings affirmed that the AHI constructed based on cardiovascular YLL could comprehensively reflect the combined effects of air pollution and ambient non-optimum temperature in Tianjin and may be sufficient for predicting the health risks associated with air pollution and non-optimum ambient temperature in the total population and different subpopulations.

Previously, the team of Professor Haidong Kan classified the AHI index into six risk levels. In this study, we classified AHI into three risk classes based on Adebajo et al [21]. In a globalized world with people traveling it is not a good idea to use entirely different risk and color schemes to communicate the risks of air pollution and non-optimal temperature.

This study has some limitations. First, while constructing an AHI based on the exposure-response relationships of air pollution and ambient temperature with health risks has the potential to better predict daily health risks for the public, it should be noted that this construction strategy and model may only apply to the Tianjin area. Future studies should incorporate data from more areas and over a more extended time frame to validate the effectiveness of this strategy. Secondly, employing monitoring data directly from stationary monitoring sites on the ground as exposure data estimates for individuals in this study inevitably introduces measurement errors and might impact the validity of the AHI. Finally, as data from other sources have not yet been obtained, this study still employed data from 2014–2017 in the validation of the AHI, which represents a limitation of this study. In future studies, it would be beneficial to include data over longer time spans and on a wider range of health outcomes, such as rates of outpatient visits, hospitalisations, and the prevalence of various health symptoms, and so on.

### Conclusion

In this work, we established an AHI based on the associations of six major air pollutants and non-optimum ambient temperature with daily cardiovascular YLL in Tianjin and evaluated the performance of the AHI in predicting daily health risks using data from 2014–2017. The AHI was found to be significantly associated with daily non-accidental and other cause-specific YLLs, and compared with the existing AQHI and AQI, the AHI had the strongest associations. And the AHI, in contrast to the AQHI and AQI, showed the strongest correlations with the risk of cause-specific YLL, both in the total population and in subpopulations. These findings highlighted that the AHI, constructed at the city level and based on cardiovascular YLL, has a greater predictive ability for health risks and has good applicability and application prospects to the public.
