# Supplementary material for: An Improved Air Health Index Based on Short-Term Cardiovascular Effects in Tianjin, China
Source: Int J Public Health. 2024 Sep 16;69:1607214. doi: 10.3389/ijph.2024.1607214 (PMC11440067; doi:10.3389/ijph.2024.1607214)
Supplement: Supplementary file 1 [file DataSheet1.pdf]

Supplemental Material

An Improved Air Health Index Based on Short-term Cardiovascular Effects in Tianjin, China

Table of Contents

The details information of DLNM

TABLE S1 Summary statistics of air pollution, meteorological factors, years of life lost in Tianjin, China, 2014-2017

TABLE S2 The increment of cardiovascular years of life lost associated with per 10 μg/m^3 increase in each pollutant at different lag days in Tianjin, China, 2014-2017

FIGURE S1 The exposure-response curve of average ambient temperature with cardiovascular YLL in Tianjin, 2014-2017, using a distributed lag nonlinear model

TABLE S3 The residual deviation of the double-threshold distributed lag linear model with varying cold and hot thresholds

TABLE S4 The distribution characteristics of air health index, air quality health index, and air quality index in Tianjin, 2014-2017

The Details Information of DLNM

We used a distributed lag nonlinear model (DLNM) to construct the exposure-response curve between ambient temperature and cardiovascular YLL. The DLNM is a prevalent statistical model used to investigate the impact of ambient temperature on health. It incorporates two functions, defining the exposure-response and lag-response relationships, respectively, to describe the intricate non-linear and lagged influences of temperature on health outcomes. Specifically, we modelled the exposure-response curve with a quadratic B-spline with three internal knots placed at the 25th, 50th, and 75th percentiles of location specific temperature distributions, and the lag-response curve with a natural cubic B-spline with an intercept and three internal knots placed at equally spaced values in the log scale. We extended the lag period to 21 days to incorporate the prolonged delay of cold effects and disregard deaths that occurred within a few days. The model is as follows:

$$E(YLL_t) = \alpha + basis.(T, lag) + \sum ns(X, 3) + ns(trend, 7 * 4) + s(rh_{lag03}) + Dow + Holiday \quad (1)$$

Where *basis.(T,lag)* is matrix obtained by applying the DLNM to ambient temperature, *T* denotes the ambient average temperature and *lag* is the lag day; *X* represents the two air pollutants with the greatest weight in the WQS, *ns(X, 3)* denotes a non-parametric smoothing function with a degree of *3* employed to regulate the effect of air pollution; *ns(trend,7*4)* denotes a non-parametric smoothing function with degree *7/year* to control for the non-linear confounding effects of long-term and seasonal trends; $s(rh_{lag03})$ is penalized spline functions to control potential confounding effect of average relative humidity; *DOW* and *Holiday* are the dummy variables to account for the short-term cyclic fluctuations in the data.

TABLE S1 Summary statistics of air pollution, meteorological factors, years of life lost in Tianjin, China, 2014-2017

Variable	Mean	SD	Min	Max	P_{25}	P_{50}	P_{75}
Air pollution							
$PM_{2.5}$(μg/m^3)	71.97	52.00	8.00	386.00	37.00	59.00	90.00
PM_{10}(μg/m^3)	116.19	72.76	11.00	866.00	67.00	98.00	143.00
SO_2(μg/m^3)	28.02	28.36	2.00	208.00	10.00	18.00	34.00
NO_2(μg/m^3)	47.88	23.12	1.60	175.00	31.00	43.00	62.00
CO(μg/m^3)	1606.44	3511.62	300.00	90000.00	920.00	1230.00	1700.00
O_3(μg/m^3)	58.04	45.94	3.00	252.00	31.00	35.00	77.00
AQI	105.45	61.65	19.36	436.96	63.00	89.00	126.09
Meteorological factors							
temperture (℃)	13.94	11.00	-16.00	34.00	3.00	16.00	24.00
Relative humidity (%)	55.37	18.43	12.00	99.00	41.50	55.00	70.00
YLLs (person-year)							
Non-accidental	1790.19	280.61	990.68	3130.39	1591.22	1776.28	1973.39
Cardiovascular	1102.27	216.05	9.38	2267.96	958.40	1081.19	1237.60
Cerebrovascular	454.44	119.52	0.00	900.21	368.93	449.31	532.57
Ischaemic	532.51	138.66	9.40	1265.40	438.35	519.60	615.40

SD: standard deviation; P_{25}: the 25th percentile; P_{50}: the 50th percentile; P_{75}: the 75th percentile

TABLE S2 The increment of cardiovascular years of life lost associated with per 10 μg/m^3 increase in each pollutant at different lag days in Tianjin, China, 2014-2017

Lag days	$PM_{2.5}$	PM_{10}	SO_2	NO_2	CO	O_3
lag0	1.17 (-1.08,3.41)	0.95 (-0.57,2.47)	4.58 (-1.09,10.25)	3.42 (-2.27,9.11)	0.09 (-0.07,0.25)	3.86* (0.72,7.00)
lag1	1.34 (-0.76,3.44)	1.58* (0.08,3.09)	10.09* (4.36,15.81)	6.25* (0.56,11.94)	0.12 (-0.03,0.27)	3.29* (0.15,6.43)
lag2	-1.88 (-3.94,0.18)	-0.80 (-2.31,0.71)	3.05 (-2.57,8.67)	1.41 (-4.15,6.97)	0.03 (-0.12,0.17)	0.62 (-2.53,3.77)
lag3	-2.24 (-4.31,-0.16)	-1.41 (-2.92,0.10)	-1.50 (-7.11,4.11)	-1.89 (-7.46,3.68)	-0.06 (-0.21,0.08)	-0.51 (-3.66,2.65)
lag01	1.81 (-0.79,4.41)	1.82* (0.01,3.63)	11.36* (4.26,18.47)	6.99* (0.15,13.82)	0.15 (-0.03,0.33)	4.34 (0.89,7.81)
lag02	0.26 (-2.70,3.22)	1.13 (-0.98,3.25)	12.91* (4.50,21.31)	6.96 (-0.83,14.75)	0.14 (-0.06,0.33)	3.84* (0.02,7.66)
lag03	-1.20 (-4.55,2.15)	0.21 (-2.21,2.63)	11.44* (1.87,21.02)	5.33 (-3.34,14.02)	0.04 (-0.18,0.26)	3.18 (-1.01,7.37)

*$P < 0.05$

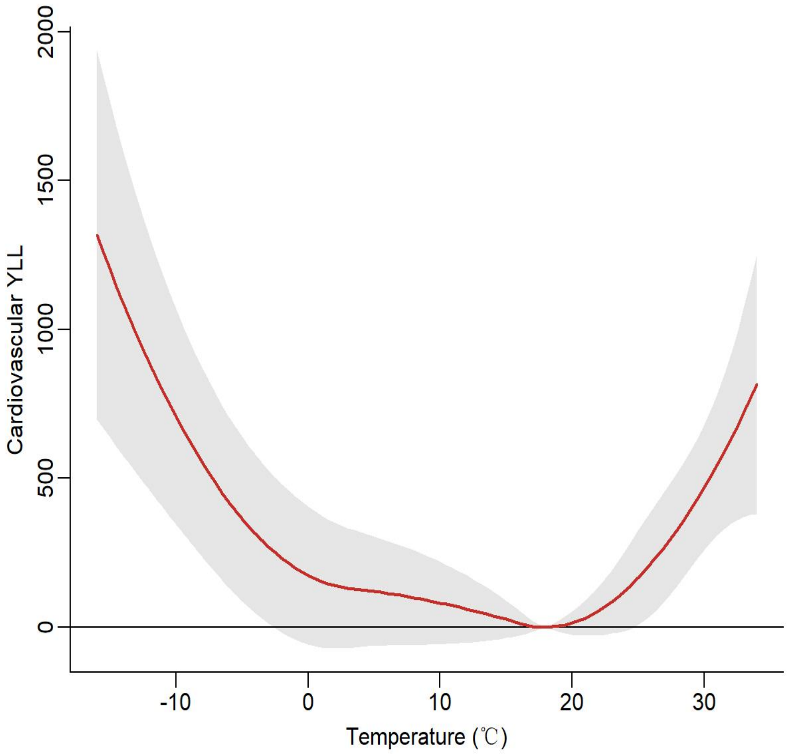

FIGURE S1 The exposure-response curve of average ambient temperature with cardiovascular years of life lost in Tianjin, 2014-2017, using a distributed lag nonlinear model

TABLE S3 The residual deviation of the double-threshold distributed lag linear model with varying cold and hot thresholds

Cold threshold \ Hot threshold	18.0℃	18.5℃	19.0℃	19.5℃	20.0℃	20.5℃	21.0℃	21.5℃	22.0℃	22.5℃	23.0℃
-3.0℃	45452763	45390722	45364612	45383155	45412762	45450888	45512838	45630963	45733070	45822923	45882169
-2.5℃	45348743	45282631	45252968	45269544	45299865	45340159	45406780	45530629	45637969	45732012	45794528
-2.0℃	45226866	45156272	45122316	45135281	45164027	45204705	45273969	45401882	45513471	45611793	45677967
-1.5℃	4508726	45013899	44976766	44986150	45011826	45051081	45120187	45248939	45362687	45464233	45534003
-1.0℃	44907917	44833698	44794999	44802004	44824676	44861589	44927707	45053441	45165886	45267391	45338189
-0.5℃	44773857	44700857	44662159	44667172	44685979	44719285	44780228	44899631	45007109	45104739	45173515
0℃	44728163	44659764	**44624128**	44629295	44645141	44674820	44728769	44837878	44935103	45022849	45084199
0.5℃	44736212	44674556	44644028	44651227	44665651	44692561	44738991	44835913	44919953	44994045	45043963
1.0℃	44808652	44755874	44732827	44744750	44760309	44786368	44826504	44911922	44982409	45041203	45076942
1.5℃	44906384	44858922	44840326	44854818	44870945	44896596	44933050	45011245	45073349	45122284	45148587
2.0℃	4500908	4496405	4494759	44963502	44980353	45006429	45042219	45118208	45177546	45222888	45245716

	2	0	6							
2.5℃	45205291	45161794	45146759	45163621	45181277	45208146	45244746	45320633	45379451	45423714	45445508
3.0℃	45332140	45286725	45269933	45285288	45302391	45329564	45368680	45448159	45511075	45559860	45586263

The smaller residuals indicate a better fit for the double-threshold DLM.

TABLE S4 The distribution characteristics of air health index, air quality health index, and air quality index in Tianjin, 2014-2017

Index	Mean±SD	Min	Max	P_{25}	P_{50}	P_{75}	*IQR*
AHI	15.60±11.13	2.47	44.61	6.34	10.67	24.49	18.15
AQHI	6.12±1.99	1.72	17.03	4.80	5.79	7.03	2.23
AQI	105.45±61.65	19.36	436.96	63.00	89.00	126.09	63.09

SD: standard deviation; P_{25}: the 25th percentile; P_{50}: the 50th percentile; P_{75}: the 75th percentile; *IQR*: inter quartile range
